# Technology-Enabled Collaborative Care for Concurrent Diabetes and Distress Management During the COVID-19 Pandemic: Protocol for a Mixed Methods Feasibility Study

**DOI:** 10.2196/39724

**Published:** 2023-01-17

**Authors:** Lenka Vojtila, Diana Sherifali, Rosa Dragonetti, Iqra Ashfaq, Scott Veldhuizen, Farooq Naeem, Sri Mahavir Agarwal, Osnat C Melamed, Allison Crawford, Philip Gerretsen, Margaret Hahn, Sean Hill, Sean Kidd, Benoit Mulsant, Eva Serhal, Leah Tackaberry-Giddens, Carly Whitmore, Jennifer Marttila, Frank Tang, Seeta Ramdass, Gloria Lourido, Sanjeev Sockalingam, Peter Selby

**Affiliations:** 1 Nicotine Dependence Service, Addictions Program Centre for Addiction and Mental Health Toronto, ON Canada; 2 Addictions Research Program, Clinical Research Centre for Addiction and Mental Health Toronto, ON Canada; 3 School of Nursing McMaster University Hamilton, ON Canada; 4 Population Health Research Institute Hamilton, ON Canada; 5 Department of Family and Community Medicine University of Toronto Toronto, ON Canada; 6 Campbell Family Mental Health Research Institute Centre for Addiction and Mental Health Toronto, ON Canada; 7 Department of Psychiatry University of Toronto Toronto, ON Canada; 8 Centre for Addiction and Mental Health Toronto, ON Canada; 9 Institute of Medical Science Temerty Faculty of Medicine University of Toronto Toronto, ON Canada; 10 Krembil Centre for Neuroinformatics Centre for Addiction and Mental Health Toronto, ON Canada; 11 Department of Psychology University of Toronto Toronto, ON Canada; 12 Vector Institute for Artificial Intelligence Toronto, ON Canada; 13 Temerty Centre for Artificial Intelligence Research and Education in Medicine (T-CAIREM) University of Toronto Toronto, ON Canada; 14 École polytechnique fédérale de Lausanne Lausanne Switzerland; 15 Department of Virtual Mental Health, Outreach and Project ECHO Centre for Addiction and Mental Health Toronto, ON Canada; 16 Diabetes Action Canada Toronto, ON Canada; 17 Aging, Community and Health Research Unit McMaster University Hamilton, ON Canada; 18 Office of Social Accountability and Community Engagement McGill University Montreal, QC Canada; 19 The Association of Faculties of Medicine of Canada Ottawa, ON Canada; 20 Conseil Pour La Protection Des Malades Montreal, QC Canada; 21 Montreal Children's Hospital Montreal, ON Canada; 22 Department of Education Centre for Addiction and Mental Health Toronto, ON Canada; 23 Bariatric Surgery Program University Health Network Toronto, ON Canada; 24 Dalla Lana School of Public Health University of Toronto Toronto, ON Canada

**Keywords:** collaborative care, type 2 diabetes mellitus, technology, mental health, diabetes, diabetic, feasibility, coaching, patient education, satisfaction, qualitative, nursing, nurse, virtual care, eHealth, digital health, health outcome, substance use

## Abstract

**Background:**

The COVID-19 pandemic disrupted the delivery of diabetes care and worsened mental health among many patients with type 2 diabetes (T2D). This disruption puts patients with T2D at risk for poor diabetes outcomes, especially those who experience social disadvantage due to socioeconomic class, rurality, or ethnicity. The appropriate use of communication technology could reduce these gaps in diabetes care created by the pandemic and also provide support for psychological distress.

**Objective:**

The purpose of this study is to test the feasibility of an innovative co-designed Technology-Enabled Collaborative Care (TECC) model for diabetes management and mental health support among adults with T2D.

**Methods:**

We will recruit 30 adults with T2D residing in Ontario, Canada, to participate in our sequential explanatory mixed methods study. They will participate in 8 weekly web-based health coaching sessions with a registered nurse, who is a certified diabetes educator, who will be supported by a digital care team (ie, a peer mentor, an addictions specialist, a dietitian, a psychiatrist, and a psychotherapist). Assessments will be completed at baseline, 4 weeks, and 8 weeks, with a 12-week follow-up. Our primary outcome is the feasibility and acceptability of the intervention, as evident by the participant recruitment and retention rates. Key secondary outcomes include assessment completion and delivery of the intervention. Exploratory outcomes consist of changes in mental health, substance use, and physical health behaviors. Stakeholder experience and satisfaction will be explored through a qualitative descriptive study using one-on-one interviews.

**Results:**

This paper describes the protocol of the study. The recruitment commenced in June 2021. This study was registered on October 29, 2020, on ClinicalTrials.gov (Registry ID: NCT04607915). As of June 2022, all participants have been recruited. It is anticipated that data analysis will be complete by the end of 2022, with study findings available by the end of 2023.

**Conclusions:**

The development of an innovative, technology-enabled model will provide necessary support for individuals living with T2D and mental health challenges. This TECC program will determine the feasibility of TECC for patients with T2D and mental health issues.

**Trial Registration:**

ClinicalTrials.gov NCT04607915; https://clinicaltrials.gov/ct2/show/NCT04607915

**International Registered Report Identifier (IRRID):**

DERR1-10.2196/39724

## Introduction

In Canada, type 2 diabetes (T2D) affects more than 1 in 10 adults. It is the single most common cause of adult-onset blindness, amputations, and kidney failure and a risk factor for cardiovascular disease, strokes, cancer, mental health disorders, and poor outcomes for all other chronic conditions [1,2]. In addition to the physical challenges, adults living with T2D are at greater risk for mental health issues than the general population. The prevalence of clinically depressive symptoms among people with diabetes is about 30%, which is 2-fold greater than that among individuals without T2D [3]. As such, a comprehensive approach to diabetes management would provide services for both physical and mental health concerns in a personalized and accessible manner [4-7].

Additionally, the negative impacts of COVID-19 on mental health and well-being among adults living with T2D are a growing concern, as this population is at greater risk for diabetes-related distress and depression [8,9]. COVID-19 has accelerated the emergence of web-based care in primary and specialist care in Canada and has resulted in the virtualization of diabetes care and support (physical and mental health) [10,11]. The rapid virtualization of diabetes care has highlighted disparities in the availability and application of digital technologies at the patient and provider level, such as having necessary resources (eg, devices, data sharing, and internet availability) and implementing telehealth effectively [12,13]. The net result is an inconsistent taxonomy and understanding of what constitutes web-based diabetes care and how to provide patient-centered care.

Currently, there is inconsistent or limited access to interprofessional diabetes education, care, and follow-up, and comorbidities such as depression, hypertension, and smoking cessation are often neglected [5,8,14-17]. Few studies have examined a web-based collaborative care model, and none, to our knowledge, have examined web-based care models addressing both mental health symptoms and physical health and diabetes management [18-22]. Overall, these programs consist of technology-mediated communications between patients and various health provider teams. However, not all interventions included a care manager or comprehensive health support (ie, psychosocial, substance use, mental and physical health, and contact with primary care or community providers). Given the greater risk that individuals with T2D will develop physical challenges and mental health issues, it is essential to build on these technological innovations and expand them to provide more comprehensive and collaborative care.

Our innovative solution will leverage a novel approach to web-based care, the Technology-Enabled Collaborative Care (TECC) model [23]. This program will provide a comprehensive approach, including a registered nurse who is a certified diabetes educator (CDE), who will support the management of individuals with T2D and mental health symptoms alongside a multidisciplinary health care team to offer health coaching for behavior change and to provide peer support. The intervention will also support people with their mental health symptoms (anxiety or depression) that may arise as a result of their diabetes diagnosis, treatment (eg, safe and effective insulin dosing schedules), or worry about access to care during the COVID-19 pandemic through one-on-one health coaching. The goal of this study is to examine the feasibility and acceptability of the TECC model designed to support patients with T2D (or the “TECC-D program”) and mental health concerns during the COVID-19 pandemic.

## Methods

The research team used the expertise of the investigative team, the CDE, research coordinators, and patient partners to co-design the format of the research study. Guiding principles include the use of existing and available technology for patients, providers, and health systems; structured and measurement-based care; collaboration across institutions and settings; integration of physical and mental health expertise; and client-centered care.

### Study Design and Workflow

This is an 8-week feasibility study. Thirty participants who meet the eligibility criteria will participate in the TECC-D program.

### Study Setting

#### Overview

This study is conducted on the internet, using readily available technologies, including REDCap (Research Electronic Data Capture; Vanderbilt University) [24] and Cisco Webex (or “Webex”). REDCap is a secure data-capturing application used to create and manage web-based surveys. Webex, a secure videoconferencing service, is used to communicate directly with participants and to support communication within the study team and the virtual care team (VCT). Participants are also given the option of using a telephone. Email is used to send participants appointment reminders and educational materials. Participants are not required to attend in-person visits, and the existing relationships with their health care providers remain unaffected. Participants are in the study for 8 weeks and followed up at 12 weeks. Participants are not reimbursed for taking part in the study.

#### Co-design With Providers

The provider-facing side of REDCap is co-designed by our CDE and principal investigators, who specialize in the clinical treatment of individuals with T2D, mental health conditions, or addiction issues. Such co-design efforts include creating a secure documenting section for post–health coaching session calls and a summary page of participant assessment data.

#### Co-design With Patient Partners

Three patient partners, who are individuals diagnosed with T2D, are involved in the research and VCT aspects of the study. On the research end, the patient partners will review research ethics board submissions, study-specific processes, phone scripts with participants, study assessment types, REDCap presentations of study assessments, and manuscript writing. Specific to the VCT, the patient partners will engage in weekly VCT rounds to discuss participant cases and provide feedback from a patient-partner perspective.

### Inclusion and Exclusion Criteria

To enhance generalizability, we use minimum exclusionary criteria. Inclusion criteria were as follows: (1) being an Ontario resident; (2) being 18 years or older; (3) having a clinical diagnosis of T2D for at least 1 year; (4) having access to diabetes education or management in the past; (5) having access to the telephone or internet through computer or mobile; (6) self-reporting experiencing some distress related to COVID-19; (7) being able to provide informed consent; and (8) being able to understand, write, and read English. Exclusion criteria included participants who are pregnant or planning to get pregnant during the course of the study (ie, in the following 12 weeks). Of note, lack of access to digital communication technology is not an exclusion criterion.

### Sample Size

This is a single-group feasibility study that aims to gather preliminary data on retention, baseline means, and variances, and examine the degree of and variability in change over time. To meet these goals, we have set the sample size at 30. Given the expected diversity of clinical presentations, patient behaviors, and comorbid mental health conditions, we believe that a moderate number of patients are necessary to allow common challenges in implementation to be identified. This sample size will also enable reasonably good characterizations of variable distributions (eg, for a variable with a mean of 50 and an SD of 10, a 95% CI for n=30 would be 46.3 to 53.7) with descriptive statistics.

### Recruitment, Consent, and Enrollment

**Figure 1:**
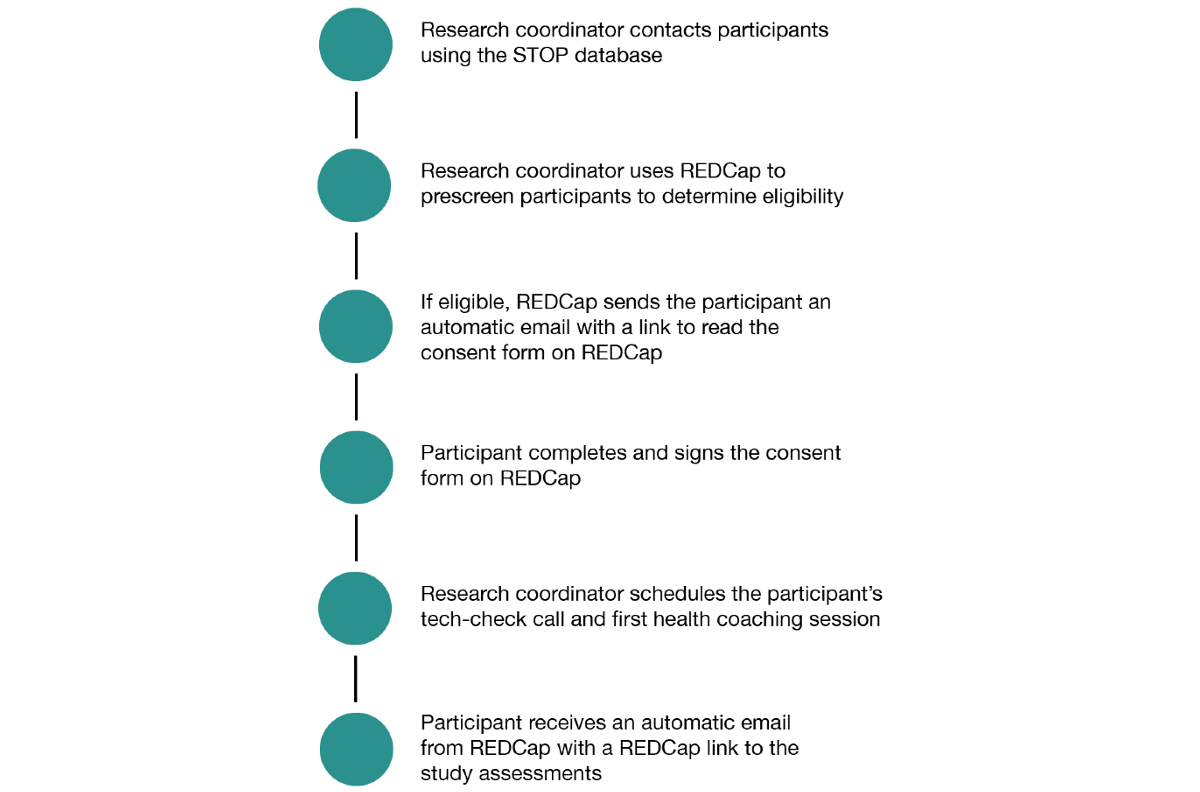
Recruitment, enrollment, and consent process via REDCap. REDCap: Research Electronic Data Capture; STOP: Smoking Treatment for Ontario Patients.

Individuals will be primarily recruited by a research coordinator via the STOP (Smoking Treatment for Ontario Patients) Program database at the Centre for Addiction and Mental Health (CAMH) in Toronto, Ontario, Canada. The STOP program delivers smoking cessation treatment to people across Ontario through various sectors, including family health teams, community health agencies, aboriginal health access centers, and addiction or mental health agencies. We will contact participants, via e-mail or telephone, who have agreed to be contacted for future research studies and who have a self-reported diagnosis of diabetes, to explore their interest in participating in this study. As most participants will likely come from the STOP Program database, our study population will include individuals who were or are daily smokers. Eligible participants will receive an automatic email to read and complete the electronic consent form via REDCap. An informational video that summarizes the consent form is provided alongside the document. Participants receive a second automatic e-mail with a link to their study assessments and 2 online follow-up appointments: (1) a technology check with a research coordinator to ensure that they are able to access REDCap and download the Webex program and (2) their first session with the CDE. Please see Figure 1 for details.

For participants who do not have access to the internet, the research team will use a verbal consent process, following the organization’s research ethics guidelines. First, the study team will send the prospective participant a hard copy of the consent form via mail. Once received, 2 research coordinators will be present during the phone call, where verbal consent will be obtained from the participant. Second, the research coordinators will complete the consent form documentation after obtaining consent and provide a hard copy to the newly enrolled participant via mail. For participants who do not have access to the internet, the study’s research coordinator will assist the participants in completing the survey and other study-related steps via telephone.

### Intervention—TECC-D Program

After providing consent, all participants will participate in the TECC-D program during their participation in the study. In this 8-week web-based collaborative care model, participants are paired with a CDE with expertise in health coaching, cognitive behavioral therapy, and motivational interviewing. Participants are asked to complete their first set of assessments before their first session. The first session (week 1) is approximately 45-60 minutes, and subsequent sessions (weeks 2-8) are 15-20 minutes. Additional 2 weeks are provided (ie, weeks 9 and 10) as an opportunity to make up for missed earlier appointments (ie, weeks 1-8). In each session, the CDE may address diabetes self-management, mental health concerns, or broader health behaviors (including diet, physical activity, smoking, alcohol use, sleep, and stress) using a client-centered approach. The role of the CDE in the participants’ care includes weekly web-based check-ins, weekly VCT rounds for individual case reviews, developing individual treatment plans, communicating with the participants’ care team (only if consent is received and as needed), and providing community resources. The CDE will help participants create goals, provide educational materials, and explore other health areas. Additionally, the CDE helps facilitate collaborative communication with the participants’ existing primary care team and may refer participants to other specialized health services. All sessions are offered via Webex or telephone.

On a weekly basis (or as needed), the CDE connects with the VCT via Webex to review the participants’ concerns and goals. The team will comprise a psychiatrist, a psychotherapist, an addictions specialist, a dietitian, and a patient partner. Other specialists (eg, an endocrinologist) will be included as needed. To facilitate asynchronous communication, the CDE uses Webex’s chat feature to send the VCT members a REDCap link to the participants’ profile and treatment summary. Here, the VCT can provide recommendations in the participants’ treatment plan synchronously during a VCT meeting or asynchronously by securely providing their recommendations in the Webex chat. Afterward, the CDE discusses the recommendations from the VCT with the participants to determine how best to implement these recommendations into their treatment plan. Participants are encouraged to communicate these recommendations to their local care providers. The intervention is articulated in Multimedia Appendix 1 [23,25,26], using the Template for Intervention Description and Replication checklist [25].

For participants who do not have access to a computer or smart device (smartphone or tablet), the research team will contact them over the telephone (landline or cell phone). As such, appointments with the CDE will be held via telephone, and if they do not have an email address, then reminder phone calls can be scheduled between the research team and the participant if needed. Any study-related material (consent forms and education materials) is sent via mail using the organization’s research ethics guidelines. For participants who have limited knowledge of technological and smart device use, the research team will provide technical support (eg, downloading applications, accessing appointments, and study documents) whenever needed.

### Institutional Collaboration

This study includes institutional collaboration between Principal Investigators and research team members from McMaster University and the CAMH, an academic specialty hospital affiliated with the University of Toronto. Essential considerations required for this study, given the institutional collaboration, include the secure transfer of data and information, privacy matters, data and study material access, abiding by the best practices and guidelines from both institutions (eg, research ethics), and finances for study-related costs.

### Program Architecture

**Figure 2:**
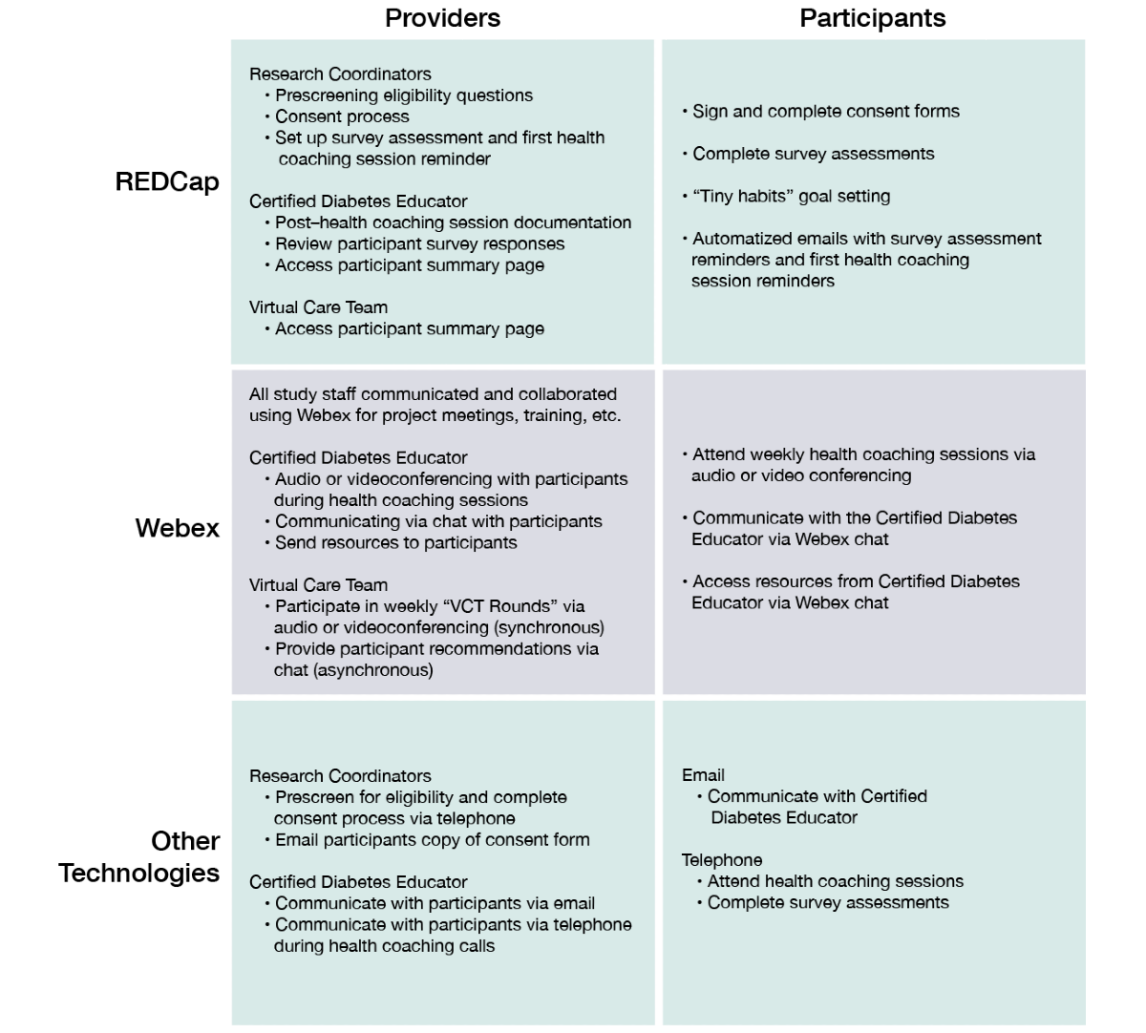
Program architecture using REDCap, Webex, and other technologies among providers and participants. REDCap: Research Electronic Data Capture; VCT: virtual care team.

With the use of REDCap and Webex, the study team is able to build a technology-based collaborative care program to enhance the experience for both participants and the study team. In summary, Figure 2 presents the use of both programs at the participant and provider levels.

### Ethics Approval

Our research is conducted in compliance with the Declaration of Helsinki [27]. This trial was approved by the CAMH in Toronto on January 18, 2021 (REB: 104-2020).

### Safety Assessment

Any adverse events will be reported and documented throughout the study period. Research staff are trained to assess for suicide risk and provide crisis interventions to participants who become distressed or endanger themselves or others. To mitigate risk, regular clinical supervision by a registered psychotherapist and a medical doctor and regular meetings with the VCT will be provided in addition to the existing care provided by the participant. Lastly, to monitor the participants’ mood, they will complete a depression scale [28] at several points throughout the study, which assesses low mood and suicidality.

### Primary Outcomes

As this is a feasibility study, we used a convenience sampling approach to assess the primary outcomes of feasibility (ie, if our intervention can be used successfully in our setting) and acceptability (ie, if our treatment is satisfactory) [29-31]. These include assessing participant recruitment (the recruitment rate [60%] is the number of participants enrolled in the study divided by the total number of eligible participants) and retention rates (the number of participants that completed the program). Key secondary outcomes include the number of participants enrolled, the number of sessions attended, and assessment completion [30-32]. Additionally, this includes how many participants chose to fill out stage 2 (“optional”) surveys, to further inform co-design with our participants for future studies. Lastly, the delivery of the intervention will be assessed, including the planned intervention sessions, the duration that the CDE was in each session, the mode of interaction (ie, whether the participant completed their visits via telephone, web-conferencing, or a combination of both), and strategies used by the CDE during health coaching sessions (eg, frequencies and examples of strategies and recommendations posed during visits), using a reporting template used in previous coaching trials [33,34].

### Exploratory Outcomes

The exploratory outcomes include clinical health outcomes related to T2D, mental health, substance use, and general health measures using valid and reliable self-reported tools. Please see Multimedia Appendix 1 for details on the rationale, procedures, and methods relating to measuring health outcomes.

### Stakeholder Experience and Satisfaction

Stakeholders, including study participants and CDE and VCT members, will have the opportunity to share their experience and satisfaction through interviews in a qualitative descriptive study. Participants will participate after the intervention, following study completion. Brief interviews will be completed one-on-one over the phone or using web conferencing technology. Each interview will be audio recorded, transcribed verbatim, and analyzed using thematic analysis. Research study members, including members of the VCT, will participate when the study is completed.

### Data Analysis

In the quantitative analysis, descriptive statistics will characterize the study sample and describe the study assessment data. This will include means, medians, ranges, and distribution.

In the qualitative analysis, a qualitative descriptive design will be used. This includes a close, comprehensive summary of experience and satisfaction using the language of the participants. All interview data will be recorded and transcribed, with close coding used to identify themes through inductive thematic analysis. One study team member will lead the analysis using Atlas.ti (version 22), with other team members contributing to organization and interpretation of identified themes.

Following the analysis of the quantitative and qualitative data, where appropriate, a mixed methods joint display will be created. This joint display will integrate the quantitative and qualitative data and help to explain the study findings more fully.

### Assessments

All participants are encouraged to complete the mandatory baseline study survey (stage 1); they have the option to complete an optional set of measures to provide more details about their health (Stage 2). This technique was used to reduce the burden of the survey on participants, as suggested by our patient partners. The first subset of assessments (stage 1) includes demographic and physical health (eg, weight and height, medications, COVID-19–related stress) questionnaires. The Diabetes Self-Management Questionnaire [35] assesses diabetes self-care activities associated with glycemic control. The Diabetes Distress Scale captures 4 critical dimensions of distress: emotional burden, regimen distress, interpersonal distress, and physician-related distress among patients with diabetes [36]. Quality of life is measured using the EuroQol-5 Dimension [37]. Tobacco use is measured using the History of Smoking Index [38]. Psychological and behavioral problems will be measured using the Global Appraisal of Individual Needs Short Screener [39].

The second subset of assessments (Stage 2) includes a visual analog scale (available in Multimedia Appendix 2) to assess the self-perceived benefit and the readiness ruler to assess the motivation and confidence of changing physical activity, nutrition, smoking, alcohol, stress, sleep, and other self-reported behaviors. Alcohol use is measured with the Alcohol Use Disorders Identification Test [40]. The Mediterranean Diet Adherence Screener is used to measure adherence to the Mediterranean diet [41]. Physical activity is measured with the International Physical Activity Questionnaire—Short Form. The Brief Pain Inventory measures pain symptoms [42]. Mental health symptoms will be measured using the Generalized Anxiety Disorder scale for anxiety symptoms [43], the Patient Health Questionnaire for depressive symptoms [28], and the Perceived Stress Scale for stress symptoms [44]. Assessment scales and tools used in this study are consistent with those employed in a previous iteration of the TECC model and supported by the study team.

## Results

At the time of submission, recruitment for this feasibility study is complete, and the remaining study participants are finishing the intervention. Data analysis is projected to commence in the fall of 2022, with the results of this study anticipated to be published by September 2023.

## Discussion

This is the first study to include a TECC program for adults with T2D (ie, the TECC-D program), including the aforementioned features, where access to digital technology by the patient is not an exclusion criterion.

The primary aim of the study is feasibility and acceptability. All participants receive the TECC-D intervention, which includes weekly health coaching calls with a CDE and personalized recommendations from a VCT. The researchers anticipate that the main finding of the study will be the feasibility of technological and collaborative health program providing diabetes- and mental health–related services to adults with T2D.

As previous studies on technology-based interventions for adults with T2D generally focused on symptom improvement and medication adherence, the goal of this study was to address other characteristics of behavioral change, including physical activity, diet, and smoking.

Additionally, previous studies have seldom engaged the individuals’ existing primary and community care teams in their collaborative care treatment.

As such, our TECC-D program puts forward a novel approach to address gaps in both research and health care among individuals with T2D. By using the existing technology in the health care system, implementing co-design of study features with practitioners and patients, and engaging patient partners with T2D, the TECC-D program illustrates a more integrated approach to collaborative care. This program aims to reduce access-related barriers to care to ensure that adults with T2D can receive the personalized, holistic care and the psychoeducation that they require.

## Appendix

Multimedia Appendix 1 TIDieR checklist.

Multimedia Appendix 2 Visual analogue scale for perceived benefit.

## Abbreviations

CAMH: Centre for Addiction and Mental Health
CDE: certified diabetes educator
REDCap: Research Electronic Data Capture
STOP: Smoking Treatment for Ontario Patients
T2D: type 2 diabetes
TECC: Technology-Enabled Collaborative Care
TECC-D: Technology-Enabled Collaborative Care for Diabetes
VCT: virtual care team

## Data Availability

This article reports the protocol of our study. No data are currently available.
