# Supplementary material for: Technology-Enabled Collaborative Care for Concurrent Diabetes and Distress Management During the COVID-19 Pandemic: Protocol for a Mixed Methods Feasibility Study
Source: JMIR Res Protoc. 2023 Jan 17;12:e39724. doi: 10.2196/39724 (PMC9890354; doi:10.2196/39724)
Supplement: Multimedia Appendix 1 [file resprot_v12i1e39724_app1.docx]

**Multimedia Appendix 1:**

**The TECC-Diabetes Program as articulated by the template for intervention description and replication (TIDieR) checklist** [23, 25, 26]

| **Item Category** | **Description** |
| --- | --- |
| *Item 1. Brief name: Provide the name or a phrase that describes the intervention* | Technology-Enabled Collaborative Care (TECC) for Concurrent Diabetes and Distress Management during COVID-19 |
| *Item 2. Why: Describe any rationale, theory, or goal of the elements essential to the intervention* | The technology-enabled collaborative care (TECC) model is a novel approach to support diabetes management. Few studies have examined a virtual collaborative care model (CCM) and none, to our knowledge, have examined virtual care models addressing both mental health symptoms, physical health and diabetes management.  In addition, the negative impacts of COVID-19 on the mental health and wellbeing among adults living with T2D are a growing concern as this population is at greater risk for diabetes-related distress and depression [11, 13]. |
| *Item 3. What (materials): Describe any physical or informational materials used in the intervention, including those provided to participants or used in intervention delivery or in training of intervention providers. Provide information on where the materials can be accessed (for example, online appendix, URL)* | The certified diabetes educator (CDE) will help participants create goals, provide educational materials, and explore other health areas. Health coaching calls are offered via Webex or telephone (based on participant’s preference). Using Webex’s chat feature, the CDE includes a REDCap link that captures the participants’ profile and treatment summary and sends it to the virtual care team (VCT). |
| *Item 4. What (procedures): Describe each of the procedures, activities, and/or processes used in the intervention, including any enabling or support activities* | The present study is being offered virtually, using several technologies including: REDCap and Cisco Webex (or “Webex”). Participants are also given the option of using telephone to communicate with the Care Manager. E-mail is also used to communicate appointment reminders and to send educational materials to participants, and to communicate with the study team. Participants are not required to attend in-person visits and existing relationships with their healthcare providers remain unaffected. Participants are in the study for 8 weeks and followed up at 12 weeks.  Individuals will be primarily recruited by a Research Coordinator (RC) from the STOP (Smoking Treatment for Ontario Patients) Program database at CAMH.  In this 8-week virtual collaborative care model, participants are paired with a Certified Diabetes Educator (CDE), with whom they communicate on a weekly basis. The first health coaching call (week 1) is approximately 45-60 minutes, subsequent calls (weeks 2-8) are 15-20 minutes. An additional two weeks are provided (i.e., week 9, 10) as an opportunity to make up for missed appointments. During these health coaching calls, the CDE addresses diabetes management, mental health, and physical health (including diet, physical activity, smoking, alcohol use, sleep and stress) with participants using a client-centered approach. The CDE will help participants create goals, provide educational materials, and explore other health areas. Additionally, the CDE helps facilitate collaborative communication with the participant’s existing primary care team and may refer the participant to other specialized health services.  On a weekly basis (or as needed), the CDE connects with the VCT via Webex to review the participant’s concerns and goals. The team will be comprised of a psychiatrist, psychotherapist, addictions specialist, dietitian, and patient partner; other specialists (e.g., endocrinologist), will be included as needed. The role of the CDE in the participant’s care includes: weekly virtual check-ins, weekly VCT rounds for individual case reviews, developing individual treatment plans, communicating with the participant’s care team (only if consent is received, and as needed), and providing community resources. To encourage engagement and motivation, the CDE uses principles of Cognitive Behavioural Therapy and Motivational Interviewing to build rapport with participants.  The provider-facing side of REDCap is co-designed by our CDE and PIs, who specialize in clinical treatment of individuals with T2D, mental health conditions or addiction issues.  The patient partners will review research ethic board submissions, study-specific processes, phone scripts with participants, study assessment types, REDCap presentation of study assessments and manuscript writing. Specific to the VCT, the patient partners will engage in weekly VCT rounds to discuss participant cases and provide their feedback from a patient-partner perspective. |
| *Item 5. Who provided: For each category of intervention provider (for example, psychologist, nursing assistant), describe their expertise, background and any specific training given* | CDE: Registered Nurse in Ontario, practicing for over 15 years, specializing in diabetes care.  Patient Partner/Peer Mentor: 3 individuals with lived experience of T2D who have experience in collaborative roles with other institutions (research/health care) via Diabetes Action Canada. They also received training on their roles as a patient partner by the Research Coordinators of the study.  Addiction Medicine Specialist and Psychiatrist: Trained and practicing physician and researcher at a mental health organization, specializing in addiction health services and psychiatry directly in the community.  Psychotherapist: Trained and practicing psychotherapist specializing in clients with substance use disorder, anxiety, and depression. Skilled provider of cognitive behavioural therapy and acceptance and commitment therapy.  Dietitian: Registered Dietitian with expertise providing nutrition and dietetics counselling to individuals living with diabetes. |
| *Item 6. How: Describe the modes of delivery (such as face to face or by some other mechanism, such as internet or telephone) of the intervention and whether it was provided individually or in a group* | Virtually, using several technologies including: REDCap and Cisco Webex (or “Webex”). During these health coaching calls, the CDE addresses diabetes management, mental health, and physical health (including diet, physical activity, smoking, alcohol use, sleep and stress) with participants using a client-centered approach. |
| *Item 7. Where: Describe the type(s) of location(s) where the intervention occurred, including any necessary infrastructure or relevant features* | Participants’ homes, access to the telephone (landline, cell phone) or internet through computer or mobile. Ontario, Canada. |
| *Item 8. When and how much: Describe the number of times the intervention was delivered and over what period of time including the number of sessions, their schedule, and their duration, intensity or dose* | Participants are in the study for 8 weeks and followed up at 12 weeks. The first health coaching call (week 1) is approximately 45-60 minutes, subsequent calls (weeks 2-8) are 15-20 minutes. An additional two weeks are provided (i.e., week 9, 10) as an opportunity to make up for missed appointments. |
| *Item 9. Tailoring: If the intervention was planned to be personalised, titrated or adapted, then describe what, why, when, and how* | Client-centered approach. On a weekly basis (or as needed), the CDE connects with the VCT via Webex to review the participant’s concerns and goals. Using Webex’s chat feature, the CDE includes a REDCap link that captures the participants’ profile and treatment summary and sends it to the VCT. The VCT will provide individualized recommendations to include in the participant’s treatment plan synchronously during a VCT meeting, or asynchronously by securely providing their recommendations in the Webex chat. Afterwards, the CDE discusses the recommendations from the VCT with the participant to determine how best to implement these recommendations into their treatment plan. |
| *Item 10. Modifications: If the intervention was modified during the course of the study, describe the changes (what, why, when, and how)* | None, protocol for a feasibility study |
| *Item 11. How well (planned): If intervention adherence or fidelity was assessed, describe how and by whom, and if any strategies were used to maintain or improve fidelity, describe them* | Participant engagement will be assessed by observing retention rates (time between first and last visit), the number of sessions attended, drop out, and study assessment completion [30, 31]. In addition, this include how many participants chose to fill out stage 2 (“optional”) surveys, to further inform co-design with our participants for future studies.  Lastly, the delivery of the intervention will be assessed including the planned coaching intervention, the amount of time the DE spent per interaction, the mode of interaction, and the strategies used during the health coaching calls by the DE. The recruitment rate (target of 60%) is the number of participants in the study divided by the total number of eligible participants. |
| *Item 12: How well (actual): If intervention adherence or fidelity was assessed, describe the extent to which the intervention was delivered as planned* | N/A, protocol for a feasibility study |
| *Revised Items - Voice: Whose voice does this description of TIDieR convey? Who was involved in the preparation of TIDieR, how they were involved in the intervention/their perspective (e.g. researcher, service deliverer, patient, etc.)?* | The research team used the expertise of PIs, CDE, collaborators, research coordinators, and patient partners to co-design the format of the research study.  The provider-facing side of REDCap is co-designed by our CDE and PIs, who specialize in clinical treatment of individuals with T2D, mental health conditions or addiction issues. Such co-design efforts include creating a secure documenting section for post-health coaching session calls and a summary page of participant assessment data.  Three patient partners, individuals diagnosed with T2D, are involved in the research and virtual care team (VCT) aspects of the research study including: review research ethic board submissions, study-specific processes, phone scripts with participants, study assessment types, REDCap presentation of study assessments and manuscript writing. Specific to the VCT, the patient partners will engage in weekly VCT rounds to discuss participant cases and provide their feedback from a patient-partner perspective. They will also host the peer group webinar. |
| *Revised Item - Stage of implementation: (i) What stage of implementation does the TIDieR checklist cover? (ii) Is this a revision of an earlier TIDieR checklist* | N/A |
| *Revised Item - How Well (actual) item; Describe the extent to which the intervention was delivered as planned and outline the factors which had an impact on actual delivery* | N/A, protocol for a feasibility study |
