# Supplementary figures and images for: Technology-Enabled Collaborative Care for Concurrent Diabetes and Distress Management During the COVID-19 Pandemic: Protocol for a Mixed Methods Feasibility Study
Source: JMIR Res Protoc. 2023 Jan 17;12:e39724. doi: 10.2196/39724 (PMC9890354; doi:10.2196/39724)

**Multimedia Appendix 2:**

**Visual Analogue Scale for Perceived Benefit**
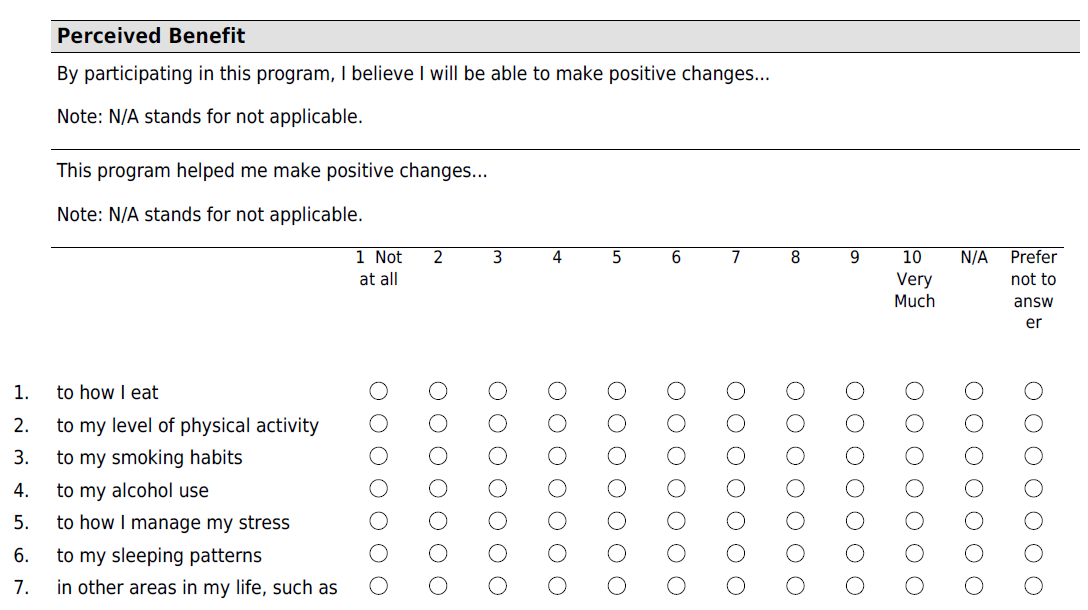

Supplement: Multimedia Appendix 2 [file resprot_v12i1e39724_app2.docx]
